# Supplementary material for: Reliability of maximal respiratory nasal pressure tests in healthy young adults
Source: PLoS One. 2023 Nov 29;18(11):e0287188. doi: 10.1371/journal.pone.0287188 (PMC10686475; doi:10.1371/journal.pone.0287188)
Supplement: S1 Table — This is the S1 Table legend n–Sample size; ICC—Intraclass correlation coefficient; SEM—Standard error of measurement; MDC—Minimum detectable change; 95% CI—Confidence interval; cmH2O - Centimeters of water. (DOCX) [file pone.0287188.s001.docx]

Supplementary Material

Table S1 presents this reliability and relation to maximum respiratory pressures, MIP and MEP. Showing good reliability of the MIP for men (ICC = 0.780) and moderate reliability for women (ICC = 0.560), with the general sample presenting good reliability (ICC = 0.870). The MEP reliability was good for women and the general population (ICC = 0.893 and 0.781, respectively); within the male population, the only low reliability was found in our results (ICC = 0.245).

**Table S1. Reliability of MIP and MEP between the highest peak and first reproducible maneuvers.**

|  | **Men**  **(n = 16)** | **Women**  **(n = 16)** | **Total**  **(n = 32)** |
| --- | --- | --- | --- |
| MIP | | | |
| ICC [95% CI] | 0.780 [0.356 - 0.924] | 0.560 [0.168 - 0.842] | 0.870 [0.734 - 0.936] |
| SEM (cmH_2_O) | 6.46 | 9.04 | 4.27 |
| MDC (cmH_2_O) | 17.9 | 25.07 | 11.85 |
| MEP | | | |
| ICC [95% CI] | 0.245 [-1.33 - 0.743] | 0.893 [0.702 - 0.962] | 0.781 [0.555 - 0.893] |
| SEM (cmH_2_O) | 17.11 | 4.29 | 7.86 |
| MDC (cmH_2_O) | 47.43 | 11.9 | 21.8 |

n – Sample size; ICC - Intraclass correlation coefficient; SEM - Standard error of measurement; MDC - Minimum detectable change; 95% CI - Confidence interval; cmH_2_O - Centimeters of water
