# Supplementary material for: Reliability of maximal respiratory nasal pressure tests in healthy young adults
Source: PLoS One. 2023 Nov 29;18(11):e0287188. doi: 10.1371/journal.pone.0287188 (PMC10686475; doi:10.1371/journal.pone.0287188)
Supplement: Attachment — Submitted filename: Response to Reviewers.docx [file pone.0287188.s003.docx]

Dear Prof. Dr. Emiliano Cè

Academic Editor

PLOS ONE

Natal, February 2022

Subject: Revision and resubmission of Manuscript PONE-D-22-15898

Dear Prof. Dr. Emiliano Cè

Thank you for revising our manuscript. We appreciate the reviewers’ complimentary comments and suggestions. We have revised the manuscript following the recommendations.

Please find attached a point-by-point response to the reviewer’s comments. We hope that you find our answers satisfactory and that the manuscript is now acceptable for publication.

Sincerely,

Prof. Dr. Guilherme Fregonezi

**Reviewer #1**

**Reviewer #1:** The scope of the review is interesting; however, I have made some remarks regarding this study.

**Introduction**

Please provide a better rationale for need to research the reliability of the SNEP and SNIP in the context of the existing knowledge on the complementarity of SNIP to MIP in the assessment of RMS. The MIP and MEP are reliable and widely used due to their simplicity, practicality, and tolerability. What is common and what differs between the methods (SNIP vs. MIP or SNAP vs. MEP)?

**Authors:** Thank you. The rationale was improved. New information and new references were added. Bellow the text.

*“Regarding the assessment of expiratory muscle strength, Morgan et al. [10] described for the first time a method similar to SNIP, the sniff nasal expiratory force. Subsequently, Ichikawa et al. [11] presented the assessment of sniff nasal expiratory pressure (SNEP). Since the technical development, the MIP and MEP tests differ from that of SNIP and SNEP from the air inlet route, the lung volume where the maneuver is carried out, the duration of the test, and possible muscle activation, therefore reliability cannot be extrapolated between tests.* **Furthermore, gender could influence SNIP and SNEP results as well as it influences other respiratory variables.** ***Preliminary data, from SNIP and MIP comparison, suggested that both tests should be used for early detection of respiratory muscle weakness to avoid over/under diagnoses [12,13].*** *The focus on precision rehabilitation requires that tests for evaluating respiratory muscle function be reliable and reproducible with each other. Tests that have low reliability and reproducibility cannot be presented as an evaluation alternative. This is why it is necessary to establish these values for these evaluations. Nevertheless, the reliability and reproducibility of SNIP and SNEP are still unknown. Therefore, this study aimed to analyze the reproducibility and reliability of SNIP and SNEP in healthy young adults.”*

**Reviewer #1:** Sample size and statistic. Did you estimate the total sample size or with differentiation of the gender? The actual and predicted value of MEP in men is different from to value in the women population.

**Authors:** Thank you for the observation. No gender differentiation was performed during sample size calculation. Gender is a relevant information for respiratory muscle strength; however, given the scarcity of research on gender differences, we opted to calculate all groups together. Due to the relevance of this information, we included it as a limitation of the study.

*“Study limitations included the small sample and a possible risk of bias (assessments and data analyses were not blind). The sample used is healthy adults, so their results cannot be directly extrapolated to the elderly population or people with neuromuscular involvement, but it is useful to leave a baseline precedent.* ***Moreover, sample size was estimated without gender differentiation, and outcome assessments and data analyses were not blinded. Therefore, results must be interpret*ed with caution, and future studies considering these limitations are needed.”**

**Reviewer #1:** Did you take into account the potential risk of losing participants in recruiting?

**Authors:** Thank you for the note. We included additional information regarding sample size calculation. Bellow is the new text with additional information.

*“Sample size was calculated using the G*Power software version 3.1.9.2 (Heinrich Heine – Universität Düsseldorf) considering the correlation between SNEP and MEP as the main variable and obtained using a coefficient of determination (r^2^) of 0.70 between SNEP and MEP variables, α of 0.001, and power (1 - β) of 0.99 and an effect size of 0.85.* ***The sample size of 14 individuals was estimated, and this number was tripled to cover possible losses, totaling the final sample of 42 subjects.”***

**Reviewer #1:** Describe what model and formula of intraclass correlation coefficient (ICC) you used.

**Authors:** Thank you for the comment. We have added information about the model used in the statistical analysis section of the manuscript.

***“Test-retest reliability was estimated using a two-way mixed-effects, type single rater and consistency model described by McGraw and Wong (1996) convention. The formula used was:***

***where: MSR = mean square for rows; MSW = mean square for residual sources of variance; MSE = mean square for error; MSC = mean square for columns; n = the number of subjects; k = the number of raters/measurements. The ICC was stratified into low (< 0.5), moderate (between 0.5 and 0.75), good (between 0.75 and 0.90), and excellent (> 0.90)”***

**Reviewer #1:** Did has calculated the coefficient of variation? Please, provide the coefficient of variation. To results section. I have proposed to assess the criterion validity, assuming the MEP, and MIP as a gold standard assessment method.

**Authors:** We did not calculate the coefficient of variation because the objective of the study was not analysis dispersion. The use of MIP and MEP to assess the criterion validity also was not the objective of the study. Thank you for the suggestion, we will take it into account in the next studies.

**Reviewer #1:** Please extend the tables with the ICC results for 10 to 20 repetitions to see the reliability of the measurements for 10, 11 to 20 repetitions.

**Authors:** Thank you for the suggestion. I am not sure we quite understand your suggestion. Table 4 showed the results for the aim os the study which was to analyze the reliability of SNIP and SNEP using the intraclass correlation coefficient (ICC) between *the highest peak pressure and the first reproducible maneuver*. In table 2 and table 3 is possible to observe the order of the highest maneuver and the first reproducible maneuver.

The results of Tables 2, 3, and 4 were described:

*Regarding Tables 2 and 3*

*“The absolute values of the highest and first reproducible maneuvers of SNIPs and SNEP. Relative and absolute frequencies showed that 60% (12 maneuvers) of the highest SNIP values and 55% (11 maneuvers) of the first reproducible occurred among the 11th and 20th maneuvers in men. For SNEP, these values were 55% and 40%, respectively. In women, 60% of the highest SNIP and 55% of the first reproducible maneuvers were also among the 11th and 20th maneuvers. SNEP measurements showed that 50% of the highest peak pressures and 60% of the first reproducible maneuvers were among the 11th and 20^th^ maneuvers.”*

*Regarding Table 4*

*“Table 4 presents the reliability among the highest peak pressures and first reproducible maneuvers stratified by sex. Table 4 shows the excellent reliability of SNIP for men and women (ICC = 0.992); the total sample also showed excellent reliability (ICC = 0.994). The reliability of SNEP was good in men (ICC = 0.877) and excellent in women (ICC = 0.957). The total sample presented an ICC of 0.950. SEM and MDC were low in both tests.”*

**Reviewer #1:** Conclusions. Present the practical implication of your results.

**Authors:** Thank you for the suggestion. We include the practical implications of our results in the conclusion.

*The ICC, SEM, and MDC were high for SNIP and SNEP, indicating their reliability in healthy young adults****. The SNIP and SNEP maneuvers are simple and inexpensive, easy to perform, and should be used as complementary to MIP and MEP to improve the diagnosis and monitoring of muscle.*** *In conclusion, following our results we suggest that twenty maneuvers were required to obtain the best results when the test is used for* ***diagnosis and monitoring of muscle weakness in several diseases in which a deficit in respiratory muscle strength is related to the natural history of the disease.***

**Reviewer #1:** You wrote: „Twenty maneuvers were required to obtain the best results without interfering with the performance of each test”. I can’t agree with it, because the results are shown only as a mean of 20 repetitions.

**Authors:** Thank you for your observation. The conclusion was based on our results:

*“We found that 75% (24 maneuvers) of the highest SNIP values and 69% (22 maneuvers) of the reproducible maneuvers were obtained in the last ten maneuvers. Also, 65% of the highest SNEP values and 62% of the reproducible maneuvers were achieved among the 11th and 20th maneuvers. According to results, respiratory muscle fatigue did not influence the tests.”*

We add new information to the conclusion to express our results.

“*The ICC, SEM, and MDC were high for SNIP and SNEP, indicating their reliability in healthy young adults.* ***The SNIP and SNEP maneuvers are simple and inexpensive, easy to perform, and should be used as complementary to MIP and MEP to improve the diagnosis and monitoring of muscle weakness.*** *In conclusion, following our results we suggest that twenty maneuvers* ***were required to obtain the best results when the test is used diagnosis and monitoring of muscle weakness in several diseases in which a deficit in respiratory muscle strength is related to the natural history of the disease.”***

**Reviewer #1:** The limitations of this study are not discussed.

**Authors:** Thank you for your suggestion. We have improved the text of the study's limitations. We hope that it covers all the limitations pointed out in the review.

*“ Study limitations included the small sample and a possible risk of bias (assessments and data analyses were not blind). The sample used is healthy adults, so their results cannot be directly extrapolated to the elderly population or people with neuromuscular involvement, but it is useful to leave a baseline precedent.* ***Moreover, sample size was estimated without gender differentiation, and outcome assessments and data analyses were not blinded. Therefore, results must be interpreted with caution, and future studies considering these limitations are needed.”***

**Reviewer #2**

This manuscript sought to investigate the reliability and sensitivity of SNIP and SNEP as the possible index to evaluate respiratory muscle strength. High to very-high ICC with low SEM% and MDC95% were found for both indexes in both sexes. The study is on the whole well written and has some potential.

The introduction is concise and the scope is clearly stated. I would only stress better the importance to differentiate the results between women and men. Although I could understand the rationale, I think it would be better to specify it better.

**Authors:** Thank you for your suggestion. We include a phrase on the rationale to express the importance of gender.

*“ Regarding the assessment of expiratory muscle strength, Morgan et al. [10] described for the first time a method similar to SNIP, the sniff nasal expiratory force. Subsequently, Ichikawa et al. [11] presented the assessment of sniff nasal expiratory pressure (SNEP). Since the technical development, the MIP and MEP tests differ from that of SNIP and SNEP from the air inlet route, the lung volume where the maneuver is carried out, the duration of the test, and possible muscle activation, therefore reliability cannot be extrapolated between tests.* ***Furthermore, gender could influence SNIP and SNEP results as well as other respiratory variables.*** *Preliminary data, from SNIP and MIP comparison, suggested that both tests should be used for early detection of respiratory muscle weakness to avoid over/under diagnoses [12,13]. The focus on precision rehabilitation requires that tests for evaluating respiratory muscle function be reliable and reproducible with each other. Tests that have low reliability and reproducibility cannot be presented as an evaluation alternative. This is why it is necessary to establish these values for these evaluations. Nevertheless, the reliability and reproducibility of SNIP and SNEP are still unknown. Therefore, this study aimed to analyze the reproducibility and reliability of SNIP and SNEP in healthy young adults.”*

**Reviewer #2:** Methods are reported. Just a suggestion: since you are proposing SNIP and SNEP as complementary indexes to assess respiratory muscle force, I would also show the correlations between SNIP and MIP and SNEP and MEP. Moreover, in the statistical analysis section, please provide some more details on the ICC approach used.

**Authors:** Thank you for the suggestion. The data from correlation will be included in another manuscript that is now in preparation with greater sample size. We have added information about the model used in the statistical analysis section of the manuscript.

“**Test-retest reliability was estimated using a two-way mixed-effects, type single rater and consistency model described by McGraw and Wong (1996) convention. The formula used was:**

**Where: MSR = mean square for rows; MSW = mean square for residual sources of variance; MSE = mean square for error; MSC = mean square for columns; n = the number of subjects; k = the number of raters/measurements. The ICC was stratified into low (< 0.5), moderate (between 0.5 and 0.75), good (between 0.75 and 0.90), and excellent (> 0.90)”**

**Reviewer #2:** Results are reported. The tables are clear.

**Authors:** Thank you.

**Reviewer #2:** Discussion: I would strongly stress here that the data are (although encouraging) from a sample of healthy and young participants. This reduces the generatability of the results to an older population or people with neurodegenerative diseases.

**Authors:** Thank you. We stress this information in the first paraph of the discussion and, is included in the study limitation text.

*“The reliability of SNIP* ***in young healthy subjects*** *was high in all contexts, ICC was close to the maximum, and SEM and MDC values were low. SNIP is reproducible, widely used [3,10,23], and recommended to assess individuals with respiratory diseases (e.g., chronic obstructive pulmonary disease and neuromuscular diseases) [24, 26]. Other studies confirmed the good reliability of SNIP for assessing healthy adults [25, 26] and children aged 8 to 11 years [28].”*

*“The reliability of SNEP* ***in young healthy subjects*** *was also good in men and excellent in women. SNEP is a novel method, and its reliability has never been studied. Morgan et al. [10] were the first to describe a nasal expiratory force in individuals with amyotrophic lateral sclerosis. In 2015, Ichikawa et al. [11] described the SNEP and showed a moderate correlation between SNEP and MEP; however, they did not assess the reliability or replicability. Unlike these authors, we demonstrated good reliability in men (ICC = 0.877) and excellent in women (ICC = 0.957); SEM and MDC were low and acceptable.”*

*“Study limitations included the small sample and a possible risk of bias (assessments and data analyses were not blind).* ***The sample used is from healthy adults so the results cannot be extrapolated directly to the elderly population or people with neuromuscular involvement,*** ***however it serves as a precedent for future clinical investigations.*** *Moreover, the sample size was estimated without gender differentiation and assessments, and data analyses were not blinded. Therefore, results must be interpreted with caution, and future studies considering these limitations are needed.”*
