# Supplementary material for: Reliability of maximal respiratory nasal pressure tests in healthy young adults
Source: PLoS One. 2023 Nov 29;18(11):e0287188. doi: 10.1371/journal.pone.0287188 (PMC10686475; doi:10.1371/journal.pone.0287188)
Supplement: Attachment — Submitted filename: Response to Reviewers.docx [file pone.0287188.s004.docx]

Dear Prof. Dr. Emiliano Cè

Academic Editor

PLOS ONE

Natal, may 2022

Subject: Revision and resubmission of Manuscript PONE-D-22-15898

Dear Prof. Dr. Emiliano Cè

Thank you for revising our manuscript. We appreciate the reviewer's complimentary comments and suggestions. We have revised the manuscript following the recommendations. Please find attached a point-by-point response to the reviewer’s comments. We hope that you find our answers satisfactory and that the manuscript is now acceptable for publication.

Sincerely,

Prof. Dr. Guilherme Fregonezi

**Reviewer #1**

For reviewer are some issues not clear describe and required clarification:

**1. Procedures
The author's wrote: The research protocol consisted of two sequences of assessments, according to simple randomization of tests. For sequence 1, participants performed inspiratory tests (MIP + SNIP1-20) followed by expiratory tests (MEP + SNEP1-20) after a 30-minute interval. Sequence 2 inverted the order of tests.” Is there a 30-minute gap between the inspiration test and the exhalation test? Was this interval between sequence 1 and sequence 2?**

**Authors:** Thank you for your question. Yes, there was a gap between sequences. We add new information to the paragraph to improve the text. Bellow the paragraph as described in the text of the manuscript procedures section:

**Procedures**

The clinical history, anthropometric, and pulmonary function data of participants were collected before MRP tests (MIP, MEP, SNIP, and SNEP). The research protocol consisted of two sequences of assessments, according to simple randomization of tests. For sequence 1, participants performed inspiratory tests (MIP + SNIP_1-20_) followed by expiratory tests (MEP + SNEP_1-20_). Between the sequences, 30-minute intervals was provide to subjects. Sequence 2 inverted the order of tests to expiratory tests (MEP + SNEP_1-20_) followed by performed inspiratory tests (MIP + SNIP_1-20_). Participants were instructed and trained to perform the maneuvers correctly. Twenty consecutive SNIP and SNEP maneuvers were performed (i.e., SNIP1-20 and SNIP1-20).

2. **The authors in response to the review wrote: “ The use of MIP and MEP to assess the criterion validity also was not the objective of the study”. Please explain which outcomes allow to formulate that conclusion:The SNIP and SNEP maneuvers are simple and inexpensive, easy to perform, and should be used as complementary to MIP and MEP to improve the diagnosis and monitoring of muscle.**

**Authors:** Dear reviewer, thanks for the opportunity to change our manuscript. We made a mistake in the response to the review. We used the MIP and MEP as a gold standard to analyze the criterion validity of SNIP and SNEP. We include in the manuscript one new table, table 5, with information about the coefficient of variation of SNIP, SNEP, MIP, and MEP values. Moreover, we add data in supplementary material with information on ICC, SEM, and MDC to MIP and MEP. The conclusions were formulated based on the results of reliability analyses. Bellow the new table add to the results section of the manuscript.

Table 5 shows the coefficient of variation (CV) of the variables SNIP, SNEP, MIP and MEP, stratified by sex. Showing the total sample in all variables moderate CV, less than 30%.

**Table 5. Table 5. Coefficient of variation of SNIP, SNEP, MIP and MEP values.**

|  | **Men**  **(n = 16)** | **Women**  **(n = 16)** | **Total**  **(n = 32)** |
| --- | --- | --- | --- |
| **SNIP** | | | |
| **Mean (cmH_2_O)** | 121.6 | 88.3 | 103.7 |
| **SD (cmH_2_O)** | 23.2 | 12.9 | 23.3 |
| **CV (%)** | 19.1 | 14.6 | 22.5 |
| **SNEP** | | | |
| **Mean (cmH_2_O)** | 131.6 | 94.9 | 111.5 |
| **SD (cmH_2_O)** | 21.8 | 22.8 | 26.5 |
| **CV (%)** | 16.6 | 24 | 23.8 |
| **MIP** | | | |
| **Mean (cmH_2_O)** | 121 | 82.2 | 101.9 |
| **SD (cmH_2_O)** | 21.7 | 19.6 | 25.4 |
| **CV (%)** | 17.9 | 23.8 | 24.9 |
| **MEP** | | | |
| **Mean (cmH_2_O)** | 128.6 | 100.8 | 115.4 |
| **SD (cmH_2_O)** | 20.2 | 33.7 | 28.7 |
| **CV (%)** | 15.7 | 33.4 | 24.9 |

SD – mean and standard deviation; CV = coefficient of variation; cmH_2_O - Centimeters of water; % - percentage

**3. In introduction the authors state: Nevertheless, the reliability and reproducibility of SNIP and SNEP are still unknown.**

In discussion section the authors wrote: „ **SNIP is reproducible**, widely used [3,10,23], and recommended to assess individuals with respiratory diseases (e.g., chronic obstructive pulmonary disease and neuromuscular diseases) [24, 26}”

**Authors:** Thanks for your note. We included the word adult in the introduction and discussion sections based on literature information. The phrase in the discussion section was modified during the process of correction and translation into English. We have included the word adult in the introduction and rephrased the sentence in the discussion section. Below is the final version:

Introduction heading:

Nevertheless, the reliability and reproducibility of SNIP and SNEP in adults are still unknown.

Discussion heading:

The reliability of SNIP in adults’ young healthy subjects was high in all contexts, ICC was close to the maximum, and SEM and MDC values were low. SNIP was considered reproducible according to our results. Moreover, SNIP is a widely used test [3,10,23],  that is recommended to assess individuals with respiratory diseases (e.g., chronic obstructive pulmonary disease and neuromuscular diseases) [24, 26]. Other studies confirmed the good reliability of SNIP for assessing healthy adults [25, 26] and children aged 8 to 11 years [28].

**4. Results: Please explain, why 17 participants were excluded from the study.**

**Authors:** This information was included in Fig. 1 of the study flowchart and is now also included in the first paragraph of the results sections.

"The sample size of 14 individuals was estimated, and this number was tripled to cover possible losses, totaling the final sample of 42 subjects. Forty-nine participants were recruited, and 17 were excluded. *From the exclusion, eight subjects  (n = 8) were excluded due to poor pressure signal  data quality), one due (n = 1) due low MEP %, six (n = 6) due low SNIP % and one (n = 1) due the value of SNEP time curve up than 500 millisecond* "

**5. Statistical analysis: Which data were parametric and which data were nonparametric?**

**Authors:**Thank you for your question. This information can be found in the footnotes of Table 1. We included this information in the results text section.

Results section first paragraph:

“ The data from age and FVC (%pred.) are considered nonparametric and expressed as median and interquartile range [25% - 75%].”

Footnotes of the table:

"Parametric data were presented as mean and standard deviation and nonparametric data as a median and interquartile range [25% - 75%]."
